# Optimized Design of Material Preparation for Cotton Linters-Based Carbon Black Dispersion Stabilizers Based on Response Surface Methodology

**DOI:** 10.3390/polym16141964

**Published:** 2024-07-09

**Authors:** Xiongfei An, Xupeng Yang, Canming Hu, Chengli Ding

**Affiliations:** College of Chemical Engineering, Xinjiang University, Urumqi 830046, China; 107552101230@stu.xju.edu.cn (X.A.);

**Keywords:** cotton linters, response surface, dispersant, carbon black

## Abstract

Carbon black particles possess dimensions on the nanometer or sub-nanometer scale. When utilized, these particles have a tendency to aggregate, which compromises their stability under storage conditions. To address this issue, a dispersant was prepared using cotton short fibers as raw materials through etherification and graft polymerization with acrylamide (AM) and 2-acrylamido-2-methylpropane sulfonic acid (AMPS) as raw materials. The dispersant was then used to disperse carbon black to test its dispersing performance. A response surface optimization test was utilized to ascertain the influence of AMPS monomer mass, AM monomer mass, and potassium persulfate (KPS) initiator mass on the dispersibility of carbon black during dispersant preparation, and a set of optimal preparation conditions were obtained. The dispersion stability of carbon black in water was assessed using Fourier transform infrared spectroscopy (FTIR), X-ray diffraction (XRD), elemental analysis (EA), thermogravimetric analysis (TG), zeta potential analysis, high magnification scanning electron microscopy (SEM), and contact angle measurements. Results revealed that the optimum mass ratio of carboxymethyl cellulose (CMC) to AMPS to AM was 1:0.69:1.67, with the KPS initiator comprising 1.56% of the total monomer mass. By incorporating the dispersant at a concentration of 37.50%, the particle size of carbon black particles was observed to decrease from 5.350 μm to 0.255 μm, and no agglomeration of carbon black particles occurred even after 3 weeks of storage.

## 1. Introduction

Carbon black is a dark powder, usually produced from heavy oil in petroleum refineries, coal, natural gas, and biomass under specific conditions. The composition of carbon black consists of discrete, nearly spherical particles, with the size of each particle varying based on the production method, often reaching the nanometer and sub-nanometer range [1]. Carbon black possesses excellent rubber reinforcement properties. Additionally, it is highly conductive and has anti-static properties. It is also used as a coloring agent and exhibits ultraviolet absorption. Due to these versatile properties, it is extensively utilized in various industries including printing and dyeing, and the production of paint, ink, rubber, plastic, foam, ceramics, silicone, leather, and cement building materials [2,3,4,5,6,7]. Even though carbon black is used in a variety of applications, it possesses a large specific surface area and exudes high surface free energy, which results in a high propensity for cluster formation and poor dispersion, making it challenging to uniformly disperse the particles within a matrix or substrate [8,9]. The technology for dispersing carbon black is pivotal for enabling practical applications of carbon black materials. Techniques such as vigorous agitation, ultrasonic treatment, and other dispersion methods have been proposed, but the dispersion of carbon black often falls short of expectations, leading to particle clumping and sedimentation [10]. Consequently, enhancing the dispersion performance of carbon black has become the focal point in this field.

Cellulose is an abundantly available polysaccharide that is composed of interconnected glucose molecules through β-1,4-glycosidic bonds [11]. Naturally available cellulose has poor utility due to its low solubility in water and vulnerability to degradation by microorganisms and enzymes [12]. Carboxymethyl cellulose (CMC) is another versatile material that has several useful properties such as water retention, along with thickening, stabilizing, and dispersing capabilities [13,14,15]. These properties arise from its relatively large specific surface area, the presence of hydroxyl functional groups, and the ability to withstand mechanical stress to a certain extent. In recent years, dispersing stabilizers based on carboxymethyl cellulose have garnered significant attention and interest in cellulose chemistry research. This is mainly due to their biodegradability, ease of chemical modification, and capacity to maintain stable dispersions of nanoparticles and emulsions, including nanosized iron tetraoxide (Fe_3_O_4_), carbon nanotubes (CNTs), graphene oxide (rGO), and others for applications in pigments, coatings, and functional composite materials [16,17,18,19].

This study selected Xinjiang’s abundant and low-cost cotton linters as raw materials. Through grafting polymerization modification to introduce functional groups such as amino and sulfonic acid groups, a high-performance waterborne dispersant was successfully prepared, enhancing the economic value of cotton linters. Utilizing response surface methodology for multi-factor analysis and response surface modeling, it optimizes multiple factors and their interactions, clarifying the effects of various process parameters on sample performance. This study determined the optimal process conditions for CMC-AMPS-AM and utilized carbon black to test dispersibility, evaluating the effectiveness of the prepared dispersant.

## 2. Materials and Methods

### 2.1. Experimental Drugs and Instruments

The cotton short staple is purchased from Aksu Prefecture, Xinjiang, China, at the Tiancheng Cotton Short Fiber Market Development Co., Ltd. (Detailed composition is listed in Table 1). Acetone (C_3_H_6_O, analytical grade), nitric acid (HNO_3_, analytical grade), urea (analytical grade), potassium persulfate (K_2_S_2_O_8_, analytical grade) were purchased in Tianjin, China, all provided by Tianjin Xinbo Chemical Co., Ltd. Anhydrous ethanol (C_2_H_5_OH, analytical grade) and sodium hydroxide (NaOH, analytical grade) were purchased from Tianjin, China, both supplied by Tianjin Zhiyuan Chemical Reagent Co., Ltd. Acrylamide (C_3_H_5_NO, Analytical grade) was procured in Tianjin, China and supplied by Tianjin Yongsheng Fine Chemical Co., Ltd. Ammonium cerium nitrate (Ce(NH_4_)_2_(NO_3_)_6_, Analytical grade) was purchased in Shanghai, China, supplied by Shanghai McLean Biochemical Technology Co., Ltd. 2-acrylamide-2-methylpropanesulfonic acid (C_7_H_13_NO_4_S, Analytical grade) was purchased from Shanghai, China, supplied by Shanghai Beide Pharmaceutical Technology Co., Ltd. Carbon Black (Analytical grade) procured in Shanghai, China, supplied by Shanghai Yien Chemical Technology Co., Ltd. Chloroacetic acid (ClCH_2_COOH, Analytical grade), procured in Tianjin, China, supplied by Tianjin BASF Chemical Co., Ltd.

The DF-101S solar collector-type magnetic heating stirrer was purchased from the Medical Instrument Factory in Jintan, Jiangsu Province, China; the SCIENTZ-10N freeze dryer was purchased from Ningbo Xinzhi Biotechnology Co., Ltd. in Ningbo, China; the BT-9300S laser particle size analyzer was purchased from Dandong Bates Instruments Co., Ltd. in Dandong, China; the QM-3SP04 planetary ball mill equipment was purchased from Nanjing ND Instruments Co., Ltd. in Nanjing, China; and the BZY-101 surface tension meter was purchased from Shanghai Jitai Electronic Technology Co., Ltd. in Shanghai, China.

### 2.2. Synthesis of CMC-AMPS-AM Hyperdispersan

#### 2.2.1. The Mechanism of Cotton Linters Staple Cellulose Modification

**Figure 1:**
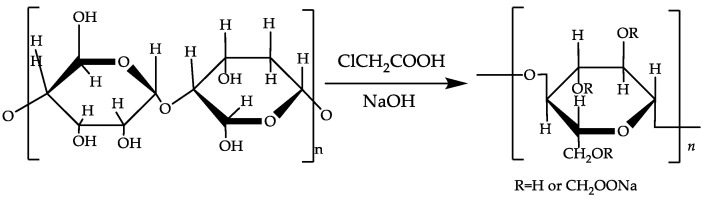
The reaction scheme for preparing CMC from cellulose.

Cellulose from cotton linters is converted into CMC via alkalinization followed by derivatization with chloroacetic acid [20,21,22]. The product is typically a white-to-grayish-white, water-soluble, odorless solid [23]. The preparation reaction scheme for CMC is shown in Figure 1.

**Figure 2:**
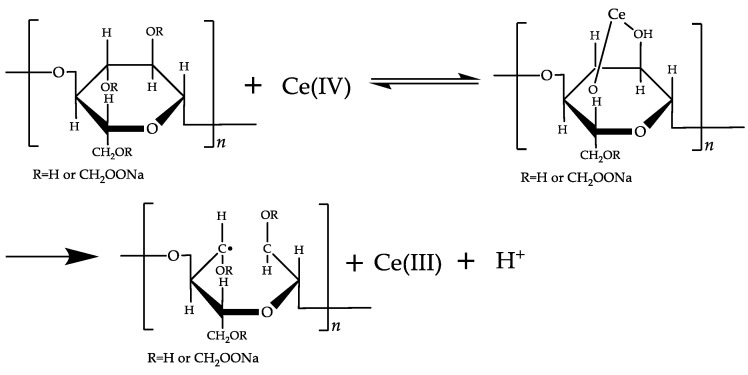
Cerium ions form complex compounds with hydroxyl groups.

Ceric ammonium nitrate (CAN) grafting mechanism [24]: During the grafting polymerization process, the acidic medium of ceric ammonium nitrate forms chelates with hydroxyl groups of cellulose, particularly between the vicinal hydroxyl groups at C2 and C3 of the glucose units in cellulose. Subsequently, through homolytic cleavage, radicals are generated on the cellulose molecular chain, opening the C2–C3 bond of the glucose ring of cellulose. This allows the cellulose to undergo free radical polymerization with the polymerizable monomers, as shown in Figure 2.

**Figure 3:**
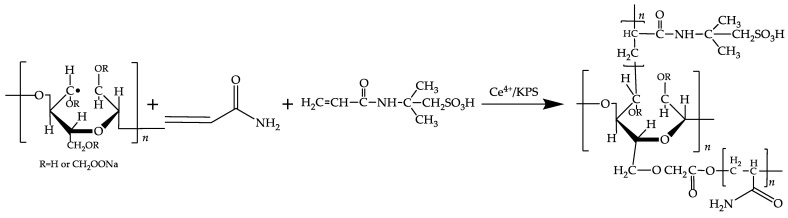
AMPS/AM modified carboxymethyl cellulose mechanism.

Using ceric ammonium nitrate [24] and potassium persulfate [25] to initiate monomer polymerization reactions, the main reactions are depicted in Figure 3.

#### 2.2.2. Sample Preparation

**Figure 4:**
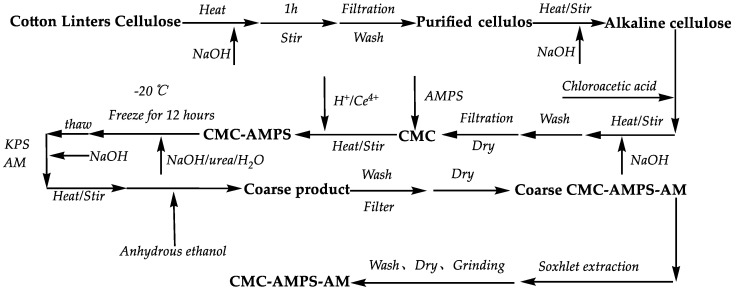
Process flow diagram for the preparation of CMC-AMPS-AM.

The cotton linters were shredded, dried, and alkalized to obtain refined cellulose, which was then etherified with chloroacetic acid to produce carboxymethyl cellulose (CMC). Then, 2 g of CMC and 0.125 mmol/L cerium ammonium nitrate solution (relative to a liquid volume of 50 mL of the reactant) were taken and the pH was adjusted to 1.0. The solution was placed in a constant temperature water bath at 50 °C and stirred for 15 min. 2-acrylamido-2-methylpropanesulfonic acid (AMPS) was slowly added to this solution, and a reaction was maintained for 4.5 h. Then, sodium hydroxide and urea were added to form a stable NaOH/Urea/H_2_O solution system (with mass fractions of 7% NaOH, 12% urea, and 81% water). The solution was frozen at −12 °C for 12 h. It was then thawed and heated to 50 °C while stirring. Potassium persulfate (KPS) and acrylamide (AM) were slowly added and reacted for 4.5 h. After the reaction, the crude CMC-AMPS-AM copolymer was precipitated and dried in double the amount of ethanol, followed by grinding. Using ethanol and acetone as solvents, pure CMC-AMPS-AM copolymer was obtained after 48 h through extraction with a Soxhlet apparatus. The preparation process is shown in Figure 4.

### 2.3. Conversion Rate Calculation

A conversion rate calculation was conducted using the elemental tracing method as shown in the following equations:

(1) $$m_{\mathrm{AMPS}}=\frac{m_{S}\times 207.250}{32}\;$$

(2) $${\mathrm{Conversion}\;\mathrm{Rate}}_{\mathrm{AMPS}}=\frac{m_{\mathrm{AMPS}}}{1.382}\times 100\%\;$$

(3) $$m_{N_{\mathrm{AMPS}}}=\frac{m_{s}\times 14.0067}{32}$$

(4) $$m_{N}{=m}_{N_{\mathrm{AMPS}}}{+m}_{N_{\mathrm{AM}}}$$

(5) $$m_{\mathrm{AM}}=\frac{m_{N_{\mathrm{AM}}}\times 71.078}{14.007}\;$$

(6) $${\mathrm{Conversion}\;\mathrm{Rate}}_{\mathrm{AM}}=\frac{m_{\mathrm{AM}}}{3.335}\times 100\%$$

Here, $m_{S}$ is the mass of sulfur in the polymer (g); 207.25 is the relative molecular mass of AMPS; 32 is the relative atomic mass of element S; $m_{\mathrm{AMPS}}\;$is the mass of AMPS in the polymer (g); 1.382 is the single cell input for AMPS (g); Con${\mathrm{Conversion}\;\mathrm{Rate}}_{\mathrm{AMPS}}\;$is the AMPS monomer conversion rate (%); $m_{N_{\mathrm{AMPS}}}$ is the nitrogen element mass from AMPS in the polymer (g); 14.007 is the relative atomic mass of element N; $m_{N_{\mathrm{AM}}}\;$is the mass of element N in the polymer from AM (g); $m_{N}$ is the total mass of nitrogen in the polymer (g); 71.078 is the relative molecular mass of AM; $m_{\mathrm{AM}}$ is the mass of AM in the polymer (g); 3.335 is the single cell input for AM (g); ${\mathrm{Conversion}\;\mathrm{Rate}}_{\mathrm{AM}}$ is the AM monomer conversion rate (%).

### 2.4. Response Surface Optimisation Experimental Design

The influential factors chosen for the response surface method were the inputs of AMPS, AM, and KPS, while the value of surface tension in the dispersant solution was considered as the response value (Y). This was then analyzed using the Box-Behnken design, which is a three-factor, three-level response surface analysis. The outcomes of this design process are documented in Table 2.

### 2.5. Description

A Fourier transform infrared spectrometer (FTIR, VEETEX-70, BRUKE, Germany) was employed for scanning within the spectral range of 400–4000 cm^−1^, with a sampling rate of 80 spectra per second. The XRD measurements were performed using a Rigaku Ultima IV diffractometer produced by Rigaku Corporation (Atsugi, Kanagawa, Japan), with a scanning range of 2θ = 10°~80°. The Bettersize 2600 laser particle size analyzer was used to measure the particle size of carbon black in the slurry. Elemental content analysis of C, H, N, and S was determined using the Vario EL cube type elemental analyzer from Germany. To obtain a uniform fine powder, the samples were subjected to a process of vacuum drying followed by grinding. Subsequently, the content of S and N was measured on the elemental analyzer. The weight loss process of the samples and the extent of heat resistance of the products were analyzed using a thermogravimetric analyzer (TA, TGA550, Milford, MA, USA). Testing conditions involved measuring the samples under a nitrogen atmosphere, with a temperature increase from room temperature to 800 °C at a rate of 10 °C/min. The contact angle between the sample and water was measured utilizing the LSA-100 (LAUDA Scientific GmbH) contact angle tester. The sample platen exerted a pressure of 10 MPa, while the observed volume of the test droplets was 2 µL. Furthermore, the appearance and morphology of the samples were observed using a scanning electron microscope (SEM, ZSISS Gemini 300, Baden-Württemberg, Germany). The Zeta potential of the sample was measured using a Zetasizer Nano ZS90 (manufactured by Malvern, UK). The testing method involved mixing carbon black and dispersant material in a ratio of 10:37.5 in ultrapure water, followed by sonication to prepare a 0.1 wt% dilute solution. Zeta potential measurements were then conducted on the dilute solution samples. The molecular weight and polymer dispersity index of dispersants materials are tested using the American Agilent 1260 Infinity Ⅱ Gel Permeation Chromatography (GPC). The test is conducted at room temperature with deionized water as the mobile phase.

## 3. Results

### 3.1. Response Surface Modelling and Analysis of Variance (ANOVA)

According to the experimental design in Table 2, the independent variables selected are AMPS (A), AM (B), and KPS (C). The experimental response value (Y) is the surface tension in the dispersant solution. The optimal process conditions are determined through response surface methodology (RSM) analysis to explore the effects among these variables, with experimental results shown in Table 3. 

Response surface test design data were designed and optimized using Design-Expert 13 software using Box-Behnken (BBD). The response value (Y) and the influence factor conformed to the quadratic response regression equation (Equation (7)).

(7) $$\begin{array}{l} Y=129.30375-4.14125\times A-41.0025\times B-194.23333\times C+0.1375\times A\times B-20.66667\times A\times C\\ -15.66667\times B\times C+0.5475\times A^{2}+6.365\times B^{2}+1038.2222\times C^{2} \end{array}$$

The model is capable of forecasting the correlation between the autonomous factors (A, B, and C) and the response variable (Y). The analysis utilized Fisher’s statistical test for the purpose of conducting an analysis of variance (ANOVA), with the outcomes of ANOVA and confidence analysis of the equations featured in Table 4. The results of the confidence analysis of the quadratic response surface regression model demonstrate that the correlation coefficient of the model fit, R^2^ = 0.9483, exceeds 0.8, indicating an excellent fit of the model with the experimental data, along with a low experimental error.

**Figure 5:**
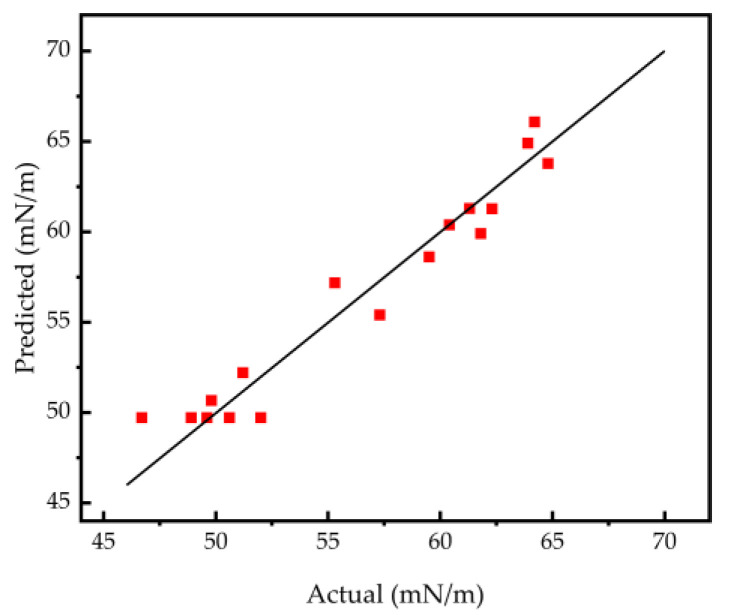
Relationship between the anticipated and actual values of solution surface tension.

The *p* value for the lack-of-fit term is 0.2205, which is greater than 0.05, suggesting that the lack-of-fit is not statistically significant [26]. Figure 5 depicts the correlation between the factual surface tension and the anticipated surface tension. The proximity in the correspondence between the projected quantities and the actual quantities signifies an elevated level of concordance between them. In this manner, it showcases the dependability of the quadratic response regression model [27].

### 3.2. Response Surface 3D Graph

**Figure 6:**
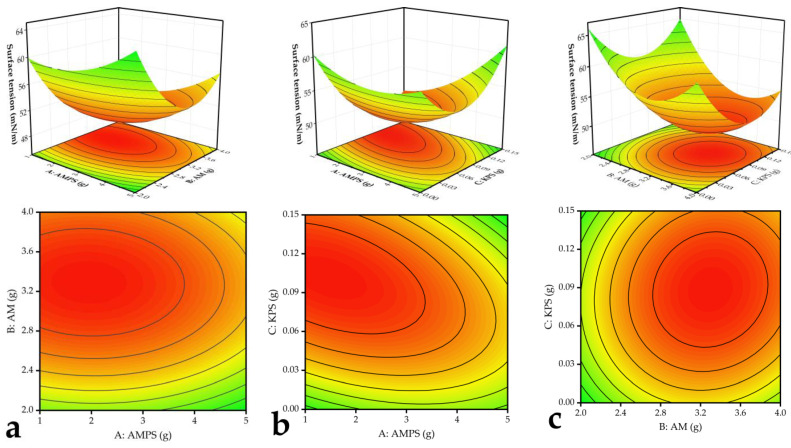
(**a**) Effect of AMPS input and AM input on dispersant surface tension. (**b**) Effect of AMPS input and KPS input on surface tension of dispersant. (**c**) Effect of AMPS input and KPS input on surface tension of dispersant.

Figure 6 depicts visualizations of the impact of each factor, namely AMPS (Figure 6a), AM (Figure 6b), and KPS (Figure 6c), on the surface tension of the solution. By observing the slope of the surface and the density of contour plots, the magnitude of the influence on the surface tension can be determined. Additionally, Table 4 provides further insight by presenting the F-values of AMPS input (A), AM input (B), and KPS input (C), which are 8.71, 22.74, and 5.53, respectively. Thus, it can be concluded that the influence of each factor on the surface tension of the solution follows this order: AM input > AMPS input > KPS input. Utilizing the proposed model, the optimization of CMC: AMPS: AM and KPS ratios resulted in an ideal composition of 1:0.69:1.67 for CMC, AMPS, and AM, respectively, with KPS being approximately 1.56% of the total monomer amount. Furthermore, the elemental content analysis revealed the N, C, H, and S contents in the samples. By employing the elemental tracing method, the sources of N and S in the CMC-AMPS-AM samples were identified, and the measurements obtained are listed in Table 5.

Based on Equations (1)–(6) in Section 2.3, the AMPS and AM grafting rates were calculated. The AMPS grafting rate was calculated to be 70.21%, and the AM grafting rate was 33.80%; the low AM grafting rate was mainly due to the polymerization of AM.

### 3.3. Carbon Black Dispersion Stability Performance

When investigating the dispersing properties of carbon black, it is crucial to take into account several pivotal factors. Firstly, it is imperative to ensure an appropriate amount of dispersant is utilized, as an insufficient quantity would not achieve the desired dispersion of carbon black, while an excessive amount would result in the formation of "bridges" by the dispersant, causing carbon black re-agglomeration. Secondly, the pH value of the dispersant also exerts a certain influence on the dispersion performance. Within an optimal pH range, the surface charge of the particles can be fine-tuned, thus facilitating the homogeneous dispersion of carbon black particles. Furthermore, the stability requirements of the dispersant should also be considered. A stable dispersant not only sustains long-term dispersion efficacy but also enhances the stability and performance of the end product.

#### 3.3.1. Effect of Dispersant Concentration

The dosage of dispersant is dependent upon the type of particles and desired particle size. In the case of pigment dispersion, the dispersant for organic pigment typically ranges from 5% to 30%, as specified by the industry. Similarly, the dispersant for inorganic pigment ranges from 5% to 20%, while for carbon black, it falls between 25% and 50%. For the high-surface-energy carbon black used in automotive coatings, such as FW 200, the recommended amount of dispersant is between 60% and 150%.

**Figure 7:**
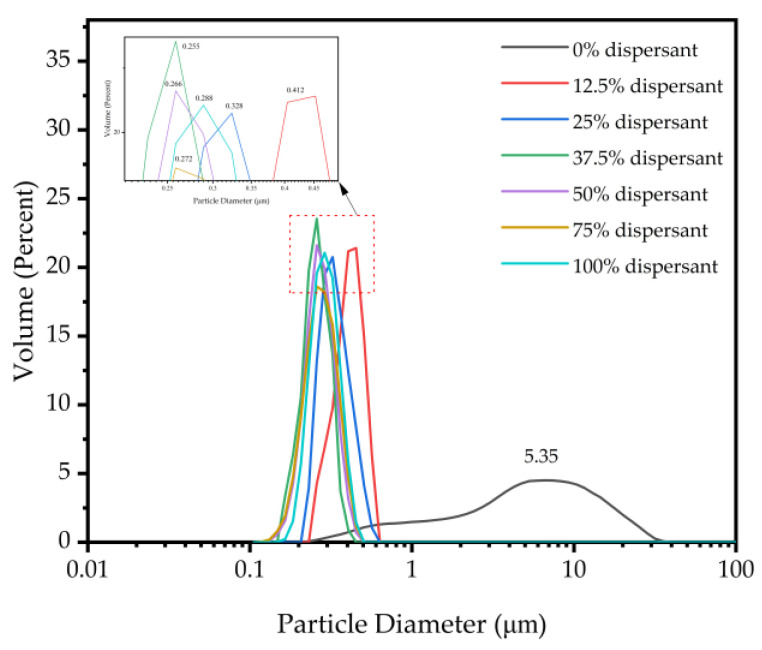
Particle size distribution of dispersed carbon black with different dispersant concentrations.

A specific quantity of dispersing stabilizer was combined with 1.5 g of carbon black, followed by the addition of 10 mL of deionized water as the solvent. Subsequently, 30 g of zirconia ball mill beads with a particle size of 0.5 mm were added, and the carbon black slurry was acquired through grinding for 5 h in a ball mill rotating at a speed of 300 rpm/min. Simultaneously, the paint without hyperdispersant was prepared as the control group, the results are shown in Figure 7. As the dispersant concentration rose from 0% to 37.5%, the size distribution of the carbon black particles shifted from a wide to narrow distribution, and the median particle size D50 decreased from 5.350 μm to 0.255 μm. Yet, when the dispersant concentration reached 100%, the median particle size D50 of carbon black particles increased to 0.288 μm. In addition, compared with other studies on carbon black dispersants, Table 6 shows that the CMC-AMPS-AM dispersant demonstrates significant advantages in enhancing carbon black dispersibility.

The agglomeration of carbon black particles is a consequence of van der Waals forces coming into play, leading to a gradual elevation in particle density and subsequent settling due to gravitational forces. The function of the dispersant is to counteract these inter-particular forces. The concentration of the dispersant directly influences the balance between dispersant molecules and carbon black particles, thereby affecting the dispersion effect. At low concentrations, insufficient dispersant coverage on the carbon black particles hinders the ability to counteract inter-particle forces. With increasing dispersant concentration, the adsorption between dispersant molecules and carbon black particles gradually rises, effectively diminishing the inter-particle interaction force and thwarting particle agglomeration, and thus resulting in reduced particle size.

#### 3.3.2. Zeta Potential Analysis

The magnitude of the Zeta potential serves as a crucial factor in assessing the stability of a dispersed system. Greater values of the zeta potential indicate enhanced electrostatic repulsion, thereby leading to superior suspension stability [31,32,33].

**Figure 8:**
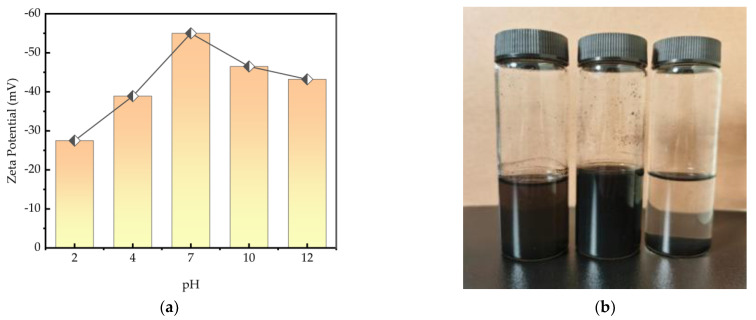
(**a**) Zeta–pH curves of carbon black and CMC-AMPS-AM. (**b**) Addition of 37.5% dispersant to carbon black at different pH (right pH = 12, middle pH = 7, and left pH = 2). Natural settling effect can be seen for the right vial with high pH.

As shown in Figure 8a, the Zeta–pH curve illustrates the dispersion of carbon black by dispersants. According to the double electric layer exclusion mechanism, charged particles adhere to the surface of particles forming a double electric layer, which results in electrostatic repulsion. In an acidic environment, there is an abundance of positive ions H+. Sulfonate ions (SO_3_^−^) readily react with H+ ions, leading to an increase in potential. Sulfate groups have a lower negative charge density, hence the Zeta potential is relatively lower in acidic environments.

In alkaline environments, a significant number of negative ions (such as OH-) are present and they interact with the positive charge on the polymer surface, causing the surface to have a negative potential. This ion exchange affects the surface potential and Zeta potential of the polymer. In an alkaline environment, the absolute value of the Zeta potential is mainly due to the presence of a large number of OH- ions in the solution, which leads to the following phenomena: (1) Charge shielding effect: A large number of negative ions, such as OH- ions, adsorb on the surface of the anionic dispersant. When the OH- concentration is sufficiently high, a charge shielding layer is formed on the surface of the dispersant material, which partially shields the negative charges, reduces the absolute value of the potential, and weakens the electrostatic repulsion. (2) Double layer thickness variation: The Zeta potential actually measures the potential difference of the double layer. When a large number of OH- ions adsorb on the surface of the anionic dispersant, the thickness of the double layer increases, which affects the transport speed of the charges, and the absolute value of the Zeta potential may decrease. (3) Solvent polarization effect: A large number of OH- ions increase the degree of solvent polarization, increase the thickness of the double layer, and weaken the electrostatic interaction between the dispersant and the solution. This reduces the absolute value of the Zeta potential. As shown in Figure 8b, the natural sedimentation effect of carbon black particles can be observed with the addition of the same amount of dispersant at different pH levels. In conclusion, CMC-AMPS-AM dispersants tend to induce agglomeration and instability of particles in strongly acidic and strongly alkaline environments.

#### 3.3.3. Stability of Carbon Black Slurry

**Figure 9:**
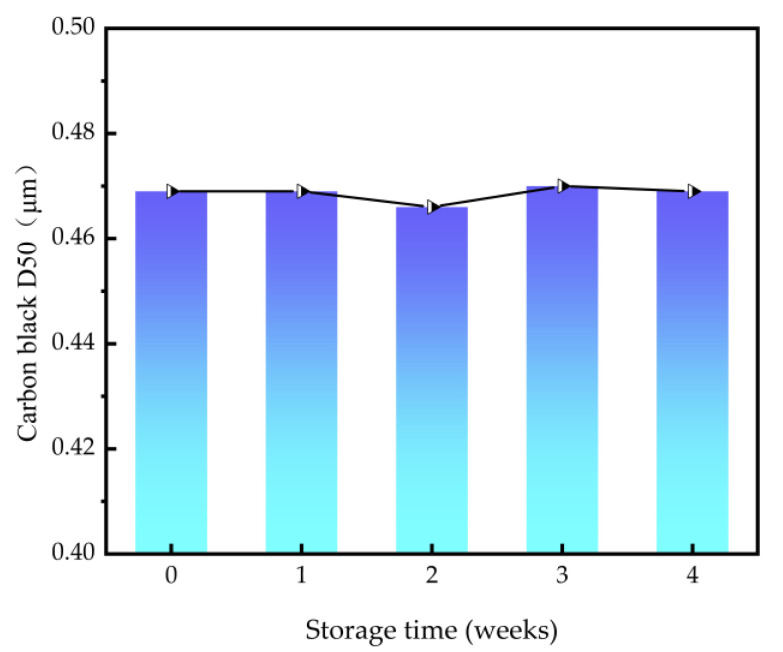
Variation of carbon black particle size with storage time.

After undergoing mechanical fracturing and abrasion with the ball mill, the particle size of carbon black decreases while the surface energy increases. This results in an elevated adhesion force between different particles that results in particle agglomeration. Hence, the stability of dispersants holds utmost significance [34,35]. In reference to Section 3.3.1 for preparing the slurry, add 10% dispersant to the carbon black, then ball mill the sample and store at room temperature. The variation in particle size is monitored every seven days. As shown in Figure 9, the particle size of the carbon black exhibits minimal fluctuation, remaining within the range of 0.469 μm before storage to 0.474 μm at the end of the fourth week. Facilitated by the dispersant, the carbon black particles surmount inter-particle adhesion as well as electrostatic forces, displaying no discernible trend of "coarsening". This suggests the dispersant confers commendable dispersion stability.

**Figure 10:**
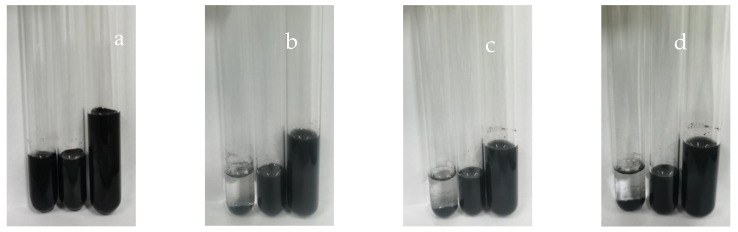
Settling of carbon black slurry in water (left bottle diluted 20 times, middle bottle diluted 80 times, and right bottle diluted 160 times): week 0 (**a**); week 1 (**b**); week 2 (**c**); and week 3 (**d**).

As shown in Figure 10, the left bottle contains carbon black slurry without dispersant added, with 0.2 g taken and diluted with distilled water 20 times as a blank group. The middle/right bottles contain carbon black slurry with 37.5% dispersant added, with 0.2 g taken and diluted with distilled water 40/160 times, respectively. The natural settling effects after 0, 1, 2, and 3 weeks are represented by a, b, c, and d. As shown in the figure, carbon black particles without dispersant coating precipitate within 7 days due to the force of attraction between molecules, causing the micro particles to aggregate into larger particles. In contrast, carbon black particles with dispersant effectively overcome the intermolecular forces between carbon black particles caused by the presence of dispersant, resulting in continuous dispersion of particles in water.

#### 3.3.4. Scanning Electron Microscopy (SEM) Analysis

**Figure 11:**
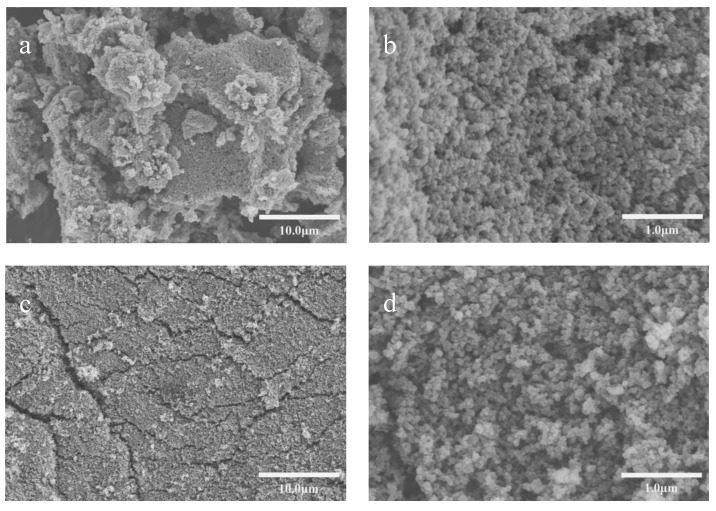
(**a**–**d**) SEM images of carbon black slurry without dispersant; (**c**,**d**) SEM images of carbon black slurry with 37.5% dispersant added.

Figure 11 depicts high-magnification scanning electron microscope images of carbon black slurries without dispersant and with 37.5% dispersant added after drying. Upon examining Figure 11a,c, it is evident that the carbon black particles, in the absence of any added dispersant, coalesce together, forming dense clusters with larger-sized particles. From Figure 11b,d, it can be observed that individual carbon black particles typically have smaller diameters, while larger particles are often formed by aggregation of smaller ones. Therefore, after the addition of dispersants, the dispersants effectively inhibit the aggregation between carbon black particles, improving the dispersibility and stability of carbon black. 

### 3.4. Characterisation of Dispersant Materials

#### 3.4.1. Infrared Spectroscopy Test (FTIR) Analysis

**Figure 12:**
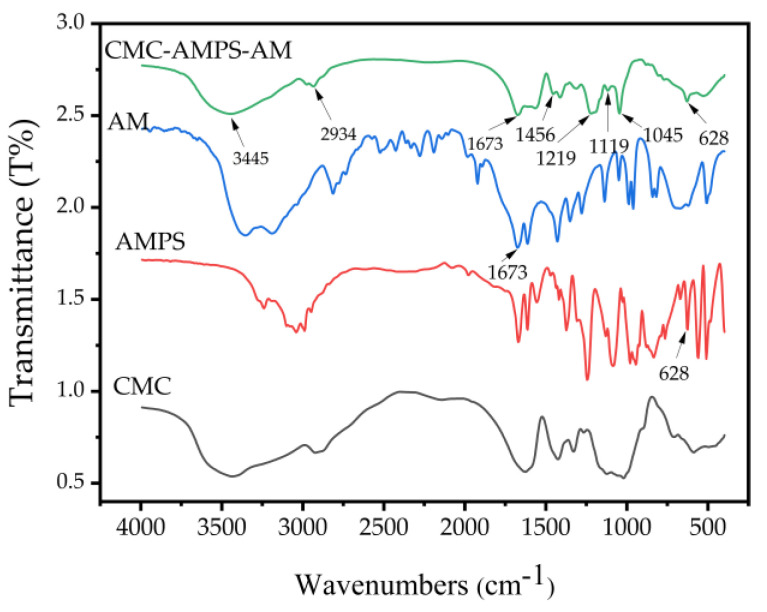
FTIR spectra of CMC/AM/AMPS and CMC-AMPS-AM.

From the FTIR spectra shown in Figure 12, it can be observed that the −NH absorption peak of the secondary amide occurred at 2934 cm^−1^, whereas the C-S stretching vibration peak in AMPS appeared at 628 cm^−1^. The -SO^3−^ in AMPS exhibited S-O bond asymmetry and S=O bond symmetry vibration absorption peaks near 1045 cm^−1^ and 1219 cm^−1^ [36]. The characteristic absorption peak at 3445 cm^−1^ is attributed to the -NH_2_ stretching vibration in AM. Furthermore, the characteristic peaks at 1673 cm^−1^ and 1119 cm^−1^ are related to the stretching vibration of the carbonyl C=O and C-N in the amide group of AM [37]. The deformation of methylene resulted in the generation of the characteristic absorption peak at 1455 cm^−1^. Through the FTIR analysis, it can be concluded that AMPS and acrylamide have been successfully grafted onto the cellulose skeleton.

#### 3.4.2. Thermogravimetric (DT) Analysis

**Figure 13:**
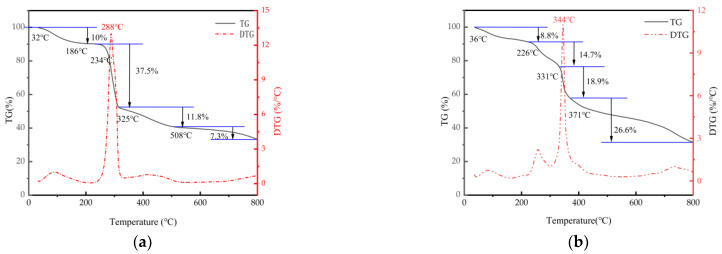
TG-DTG curve of (**a**) CMC and (**b**) CMC-AMPS-AM.

TG-DTG curves illustrate the weight loss of CMC and CMC-AMPS-AM, as shown in Figure 13. For CMC (Figure 13a), it is discernible that before the temperature reaches 32 °C–234 °C, there is a relatively insignificant weight loss of approximately 10%, which is attributed to the removal of adsorbed water on the carboxymethyl cellulose surface. Between 234 °C and 325 °C, there is a prominent weight loss of about 37.5%, stemming from the rupture and decay of C=O, C-O-C, and -OH bonds. Notably, the thermal degradation of cellulose attains a maximum when the temperature peaks at 289 °C, primarily due to a substantial number of C–C bond ruptures, leading to a vast mass reduction in cellulose. Throughout the entire process, spanning from 32 °C to 800 °C, the overall weight loss accounts for approximately 66.6% of the mass.

In the case of CMC-AMPS-AM (Figure 13b), a negligible reduction in mass of the CMC-AMPS-AM copolymer is observed within the temperature interval of 36 °C to 100 °C. This phenomenon can be attributed to the low amount of moisture adhered to the CMC-AMPS-AM copolymer, as well as the weight decrease caused by the evaporation of acetone. In contrast, the weight loss percentage of carboxymethyl cellulose is approximately 14.7% within the temperature range of 226 °C to 331 °C and 37.5% within the range of 234 °C to 325 °C. This indicates a considerable decline in weight loss in CMC, likely due to the grafting reaction that disrupts the cyclic structure in the sugar component and imparts greater stability. In the temperature range of 331 °C to 371 °C, the weight loss was approximately 18.9%, possibly due to the thermal decomposition of the amide group in AM and the subsequent imidization reaction. Throughout the temperature range of 32 °C to 800 °C, the weight of the CMC-AMPS-AM copolymer witnessed a total decrease of approximately 69%.

#### 3.4.3. X-ray Diffraction (XRD) Analysis

**Figure 14:**
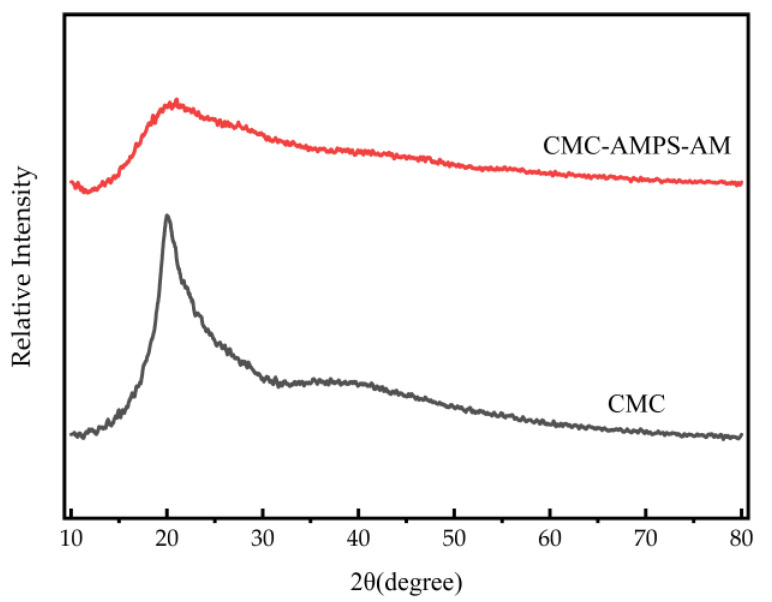
XRD spectra of CMC-AMPS-AM and CMC.

XRD results for CMC and CMC-AMPS-AM samples are shown in Figure 14, where it can be observed that in the grafting of AMPS, AM could impact the chemical reaction and molecular structure, subsequently causing deviations in the crystal structure, diminished grain size, and defects in the cellulose crystals. As a result of the decreased grain size and crystallinity, the peaks in the XRD pattern became broader, with a rounded shape and lower intensity.

#### 3.4.4. Molecular Weight Testing

According to the dispersion mechanism of dispersants, the molecular weight of dispersants is closely related to their dispersing performance. Gel permeation chromatography (GPC) tests were conducted on CMC-AMPS-AM dispersant materials, and the test results are shown in Table 7.

#### 3.4.5. Contact Angle Analysis

**Figure 15:**
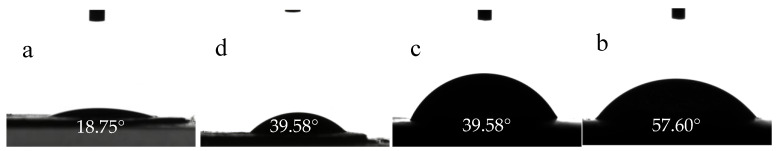
Contact angle between material and water: (**a**) CB; (**b**) CMC; (**c**) CMC-AMPS-AM; and (**d**) CMC-AMPS-AM and CB.

The dispersant composition not only affects the dispersion of pigment particles but also affects the surface energy of the resulting dry pigment particles. The smaller the contact angle, the higher is the hydrophilicity of the particles [38,39]. After mixing carbon black with CMC-AMPS-AM dispersant material in a 1:1 ratio, measure the contact angle with water. As shown in Figure 15, the untreated carbon black surface is rich in polar functional groups such as hydroxyl carbonyl groups, which attracts hydrogen atoms of water molecules, rendering it hydrophilic. Carboxymethyl (-CH_2_COOH) functional groups were prepared through etherification of cellulose. Due to the grafting of AMPS and AM, the concentration of -COOH groups in carboxymethyl cellulose decreased, the contact angle increased, and the hydrophilicity decreased. The contact angle increased after adding CMC-AMPS-AM to carbon black. In the application of carbon black coatings, highly hydrophilic carbon black particles are not compatible with waterborne coatings, and also not conducive to the water resistance of coatings [40,41].

### 3.5. Dissemination Mechanisms

**Figure 16:**
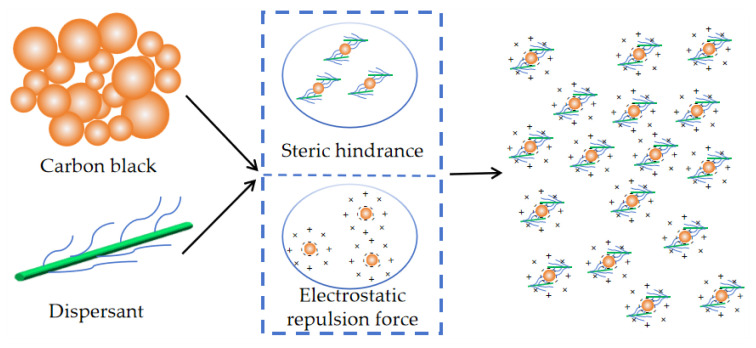
Dispersion agent dispersion mechanism.

According to the dispersant dispersing mechanism, dispersants mainly rely on steric effects and electrostatic interactions [42,43,44,45]. Steric hindrance: dispersant molecules or ions adsorb on the surface of solid particles, forming physical barriers that hinder direct contact and aggregation of particles. Electrostatic repulsion force refers to the repulsive force generated by electrostatic interaction between particles of the same charge, effectively preventing particle aggregation and settling, promoting particle dispersion, and maintaining suspension stability. In practical dispersion, both mechanisms work synergistically [46]. The CMC-AMPS-AM material disperses carbon black as shown in Figure 16, achieving optimized dispersion through the synergistic effects of steric hindrance and electrostatic repulsion force.

## 4. Conclusions

Carboxymethyl cellulose is derived through the process of etherification, using the abundant resource of cotton linters. Tetravalent cerium ions have the ability to engage in redox reactions with various reducing groups, such as hydroxyl groups, ultimately producing free radicals. These radicals serve as catalysts for grafting reactions with acrylamide monomer and 2-acrylamide-2-methylpropanesulfonic acid monomer. By employing the response surface method, the optimal values for three influential factors were determined. The initiator KPS comprised 1.56% of the total amount of monomers, and the ideal ratio of raw materials CMC:AMPS:AM was established as 1:0.69:1.67. The order of the impact of these factors on the conversion rate was AM > AMPS > KPS. Through elemental tracing, the contents of S and N were determined under the optimal preparation process. Consequently, the elemental tracking method enabled the determination of S and N contents, and the conversion rates of AMPS and AM were determined to be 70.21% and 33.80%, respectively. Upon application of the CMC-AMPS-AM dispersant to carbon black materials, the median particle size (D50) of the carbon black decreased to 0.255 μm. Moreover, the distribution of particle sizes became more concentrated as the amount of dispersant additive reached 37.5%. Notably, even after a storage period of four weeks, the particle size of the carbon black remained stable, without exhibiting the so-called “re-coarsening” phenomenon.

In the optimization of dispersant synthesis process, there are challenges in terms of process complexity and long cycles, which pose a significant challenge for industrial production. The stability assessment of dispersants does not consider performance under extreme conditions, such as high and low temperatures. Additionally, industrial applications often require compatibility with various additives such as ink additives, coating resins, and tire rubber, but this study does not cover their compatibility and synergistic effects. Since cellulose is insoluble in water, etherification treatment is required to prepare water-based dispersants, converting it into carboxymethyl cellulose, which increases costs and industrial difficulty. Cotton short fiber cellulose has a high content and is easy to extract, but most biomass cellulose content in nature is low. The future direction of research is to use low-cellulose biomass for modification and directly prepare water-based dispersants.

## Figures and Tables

**Table 1:** Cotton linters composition and content.

Component Name	Cellulose	Pectin	Wax	Lignin	Ash Content
content/%	>95	0.5	1	1~2	1

**Table 2:** Response surface experimental design.

Level	Considerations		
	A:AMPS (g)	B:AM (g)	C:KPS (g)
−1	1	2	0
0	3	3	0.075
1	5	4	0.150

**Table 3:** Response surface experimental design and results.

Std	AMPS	AM	KPS	Surface Tension of Solution (mN/m)
16	0	0	0	48.9
15	0	0	0	52.0
4	1	1	0	55.3
17	0	0	0	49.6
5	−1	0	−1	60.4
7	−1	0	1	49.8
9	0	−1	−1	64.2
3	−1	1	0	51.2
1	−1	−1	0	61.8
14	0	0	0	50.6
2	1	−1	0	64.8
12	0	1	1	57.3
6	1	0	−1	59.5
10	0	1	−1	62.3
8	1	0	1	61.3
13	0	0	0	47.5
11	0	−1	1	63.9

**Table 4:** Analysis of variance of regression equations and credibility of regression model.

Source	Sum of Squares	df	Mean Square	F-Value	*p*-Value	Significance
Model	576.94	9	64.10	14.26	0.0010	Significant
A-AMPS	39.16	1	39.16	8.71	0.0214	
B-AM	102.25	1	102.25	22.74	0.0020	
C-KPS	24.85	1	24.85	5.53	0.0510	
AB	0.3025	1	0.3025	0.0673	0.8028	
AC	38.44	1	38.44	8.55	0.0222	
BC	5.52	1	5.52	1.23	0.3043	
A²	20.19	1	20.19	4.49	0.0718	
B²	170.58	1	170.58	37.94	0.0005	
C²	143.60	1	143.60	31.94	0.0008	
Residual	31.47	7	4.50			
Lack of Fit	19.88	3	6.63	2.29	0.2205	not Significant
Pure Error	11.59	4	2.90			
Cor Total	608.41	16				

**Table 5:** Elemental analyzer measurement data table.

Specimens	N (%)	C (%)	H (%)	S(%)
CMC-AMPS-AM	4.77	33.67	5.46	2.69

**Table 6:** Comparison of the effects of different dispersants on the particle size of carbon black.

Dispersed Materials	D50 Particle Size of Carbon Black (μm)	Reference
DP-3537	1.80	[28]
IMD	0.275	[29]
PIB	0.100	[30]
BYK-192	0.785	[29]

**Table 7:** The results of the GPC test for the CMC-AMPS-AM dispersant material.

Specimens	Mn	Mw	PDI
CMC-AMPS-AM	148159	278299	1.878381

## Data Availability

Data are contained within the article.
